# Exploring the potential value of miR-148b-3p, miR-151b and miR-27b-3p as biomarkers in acute ischemic stroke

**DOI:** 10.1042/BSR20181033

**Published:** 2018-11-28

**Authors:** Xiuli Cheng, Pengcheng Kan, Zhuolin Ma, Yaru Wang, Wen Song, Chao Huang, Biao Zhang

**Affiliations:** 1 Department of Clinical Laboratory, Tianjin Key Laboratory of Cerebral Vessels and Neural Degeneration, Tianjin Neurosurgical Institute, Tianjin Huanhu Hospital, Tianjin, China; 2 Graduate School, Tianjin Medical University, Tianjin, China

**Keywords:** biomarker, circulating microRNA, diagnosis, ischemic stroke

## Abstract

Cerebrovascular disease is the main cause of death in the world. Here, we explored whether circulating serum miR-148b-3p, miR-151b and miR-27b-3p could be as potential diagnostic biomarkers for diagnosing acute ischemic stroke. Seventy-seven IS patients and forty-two healthy controls matched for age and sex were enrolled in the present study. Blood samples were drawn from IS patients within the 24 h. The correlation analysis was performed by Spearman. The ability to distinguish patients from healthy controls was determined by receiver operating characteristic (ROC) curve. The expression of circulating serum miR-148b-3p was significantly decreased, whereas miR-151b and miR-27b-3p were elevated significantly compared with controls. ROC analysis showed area under the ROC curve (AUC) of miR-148b-3p, miR-151b and miR-27b-3p to be 0.6647, 0.6852 and 0.6657, respectively. While the AUC increased to 0.8103 for the combination of miR-148b-3p and miR-27b-3p. Blood miR-151b level was negatively correlated with insulin-like growth factor-1 (IGF-1), and miR-27b-3p level was negatively correlated with IGF-1 and insulin-like growth factor binding protein-3, respectively. Our findings suggest that miR-148b-3p, miR-151b and miR-27b-3p may serve as blood-based biomarkers for diagnosing ischemic stroke patients, and the combination of miR-148b-3p and miR-27b-3p may be more powerful.

## Introduction

Currently, cardiovascular and cerebrovascular disease became the two leading cause of years of life lost, and in China, the leading cause of death in 2016 was cerebrovascular disease for men and women [1]. Ischemic stroke (IS) is the most general type of cerebrovascular disease that occurs when the brain–blood circulation is interrupted by a clot blocking a blood vessel. Unlike myocardial infarction, until recently, there are still no current blood biomarkers to predict and diagnose ischemic stroke. The diagnosis today mainly based on clinical examinations, confirmed with magnetic resonance imaging (MRI) or computed tomography (CT), which are time-consuming and expensive. So, it is urgent to develop serum/plasma biomarkers for accurate prediction and diagnosis of ischemic stroke.

MicroRNAs (miRNAs or miRs) are a group of small (19–23 nt) non-coding single strand RNA molecules that play endogenous roles in regulating gene expression through binding to 3′-untranslated regions of target mRNAs [2]. It is well known that miRNAs have become the important regulators and carry out key roles in different pathophysiological conditions, such as proliferation, differentiation, migration, secretion, cell cycle and apoptosis [3]. Up to date, lots of reports have demonstrated that specific circulating miRNAs are highly stable and detectable in serum/plasma from patients with different diseases, including ischemic stroke. This unusual stability suggests that circulating miRNAs could serve as revolutionary potential biomarkers for various kinds of diseases diagnosis [4]. Several studies have found circulating miRNAs change following stroke in humans, either up-regulated or down-regulated, and revealing that they might be promising biomarkers for the detection of stroke [5]. Although many circulating serum miRNAs as biomarker candidates have been reported to be valuable in ischemic stroke, none of them has been widely used in clinical practice [6]. The main reason is that the methodology is not standardized and the results are not comparable as well as the cutoff values for diagnosis of IS are not clearly determined.

In previous study, we screened the differential expression profile of circulating miRNAs in patients with ischemic stroke and healthy controls using Agilent miRNA array (V18.0) (Agilent Technologies, Inc, Santa Clara, CA, U.S.A.) [7]. We found that miRNA-148b, miRNA-151b and miRNA-27b-3p were significant difference between patients with ischemic stroke and healthy controls. In the present study, we explored circulating serum levels of miR-148b-3p, miR-151b and miR-27b-3p in IS patients and healthy controls, analyzed the correlations between these circulating serum miRNAs levels and clinical characteristics. The receiver operating characteristic (ROC) curve was introduced to assess the ability of these serum miRNAs in distinguishing the IS patients and healthy controls, and the area under the ROC curve (AUC) was calculated, aiming to determine whether circulating serum levels of miR-148b-3p, miR-151b and miR-27b-3p are potential biomarkers for diagnosing ischemic stroke.

## Materials and methods

### Patients and healthy controls

This research was confirmed by the Ethics Committee of Tianjin Huanhu Hospital. Written informed consents were acquired from all the volunteers. A total of seventy-seven patients were enrolled in the present study with a mean age of 61 years (range, 35–79 years). All subjects were admitted to the emergency department less than 6 h after the onset of symptoms and diagnosed by CT or MRI scanning. Forty-two healthy volunteers without history of cerebrovascular diseases were recruited from the physical examination center as healthy controls. They were age and sex matched with IS patients. All subjects and healthy controls were excluded from cancer.

### Serum preparation

The blood samples of ischemic stroke patients were drawn within 24 h after their admission to the hospital. And all healthy control samples were collected fasting 8–12 h. Blood was centrifuged at 3000 rpm for 15 min, and serum was maintained at −80°C until measurement.

### RNA isolation and qRT-PCR detection

Total miRNA was isolated and purified from 200 μl serum by miRNA Purification Kit (Catalog No.CW0627, CW Biotech, Beijing, China) following the manufacturers’ instructions. MiRNA cDNA was synthesized through total RNA reverse transcription following the manufacturers’ protocol using miRNA cDNA Synthesis Kit (Catalog No.CW2141, CW Biotech, Beijing, China). Expressions of miRNAs were carried out using SYBR green-based quantitative real-time PCR using miRNA quantitative PCR Kit (Catalog No.CW214, CW Biotech, Beijing, China) with the CFX96 real-time PCR instrument (BioRad, Hercules, CA, U.S.A.). Small nucleolar RNA U6 served as the endogenous control to avoid technical and experimental variations among the healthy and stroke samples. Specific primers for each miRNA were obtained from the Sinogene Biotech. The relative expression levels of miR-148b-3p, miR-151b and miR-27-3p were calculated and expressed as 2^-∆*C*t^, where ∆*C*_t_ = cycle threshold (*C*_t_) (miRNA) − *C*_t_ (U6).

### Statistical analysis

The one-sample Kolmogorov–Smirnov test was introduced to evaluate the normality of data distribution. Continuous variables were presented as mean ± SD or median (25–75th quartile), and categorical variables were presented as frequency. Differences in clinical parameters between patients and healthy control were determined by Student’s *t* test for normal distribution data, the Mann–Whitney *U* test for skewed data or the Chi-Square test for categorical variables. The ability to distinguish the IS patients and healthy controls was determined by ROC curve, and AUC was calculated. Correlations between serum levels of miRNAs and laboratory determinations were analyzed by the Spearman correlation test. All statistical data were analyzed by the SPSS 20 software and GraphPad Prism 5. *P*<0.05 was considered statistically significant.

### Ethics approve and consent to participate

The study was approved by the Ethics Committee of Tianjin Huanhu Hospital. Written informed consent was provided in accordance with the Declaration of Helsinki.

### Availability of data and materials

The datasets used and/or analyzed during the current study are available from the corresponding author on reasonable request.

## Results

### General parameters of the participants

The general parameters of the IS patients and controls are shown in Table 1. Serial of parameters, including age, gender, total triglyceride, total cholesterol, HDL-C, LDL-C, glucose, smoking, drinking, hypertension, diabetes and NIHSS were recorded. No significant differences were found among IS patients and healthy controls except hypertension.

**Table 1:** General clinical characteristics of ischemic stroke patients and healthy controls

Characteristics	IS (*n*=77)	Control (*n*=42)	*P* value
Age (years)	61 ± 10.4	59 ± 4.7	>0.05
Gender (M/F)	54/23	29/13	1.000
TG (mmol/l)	1.52 ± 0.94	1.50 ± 0.85	0.905
TC (mmol/l)	4.79 ± 1.19	5.06 ± 0.73	0.198
HDL-C (mmol/l)	1.14 ± 0.24	1.21 ± 0.29	0.154
LDL-C (mmol/l)	2.85 ± 1.00	3.15 ± 0.59	0.074
Glucose (mmol/l)	6.10 ± 2.50	5.43 ± 0.84	0.092
Smoking, *n* (%)	29 (37.66)	15 (35.71)	0.833
Drinking, *n* (%)	23 (29.87)	11 (26.19)	0.671
Hypertension, *n* (%)	54 (70.13)	20 (47.62)	0.015*
Diabetes mellitus, *n* (%)	15 (19.48)	6 (14.29)	0.478
NIHSS	7.62 ± 5.75	–	–

Abbreviations: M, male; F, female; TG, total triglyceride; TC, total cholesterol; HDL-C, high-density lipoprotein cholesterol; LDL-C, low-density lipoprotein cholesterol; NIHSS, national institute of heath stroke scale; *P<0.05.

### Serum miRNAs levels in IS patients and healthy controls

**Figure 1:**
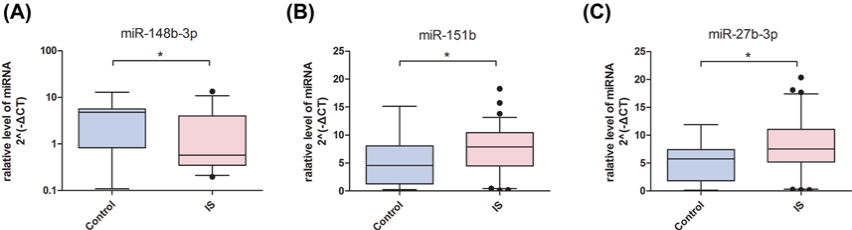
Circulating serum levels of miR-148b-3p, miR-151b and miR-27b-3p in ischemic stroke patients (*n*=77) and healthy controls (*n*=42) The whiskers of the plots represent the 5 to 95 percentiles. The lower, middle and upper lines of box represent the lower quartile, median and the upper quartile, respectively. The points represent outliers; *P<0.05.

We detected the circulating serum levels of miR-148b-3p, miR-151b and miR-27b-3p in IS patients and healthy controls using qRT-PCR assays. As showed in Figure 1, the expression of miR-148b-3p was significantly decreased in ischemic stroke patients (*P*<0.05) (Figure 1A), whereas the levels of miR-151b (Figure 1B) and miR-27b-3p (Figure 1C) were up-regulated significantly compared with healthy controls (both *P*<0.05).

### Correlation of serum miRNAs expression levels with clinical characteristics

**Figure 2:**
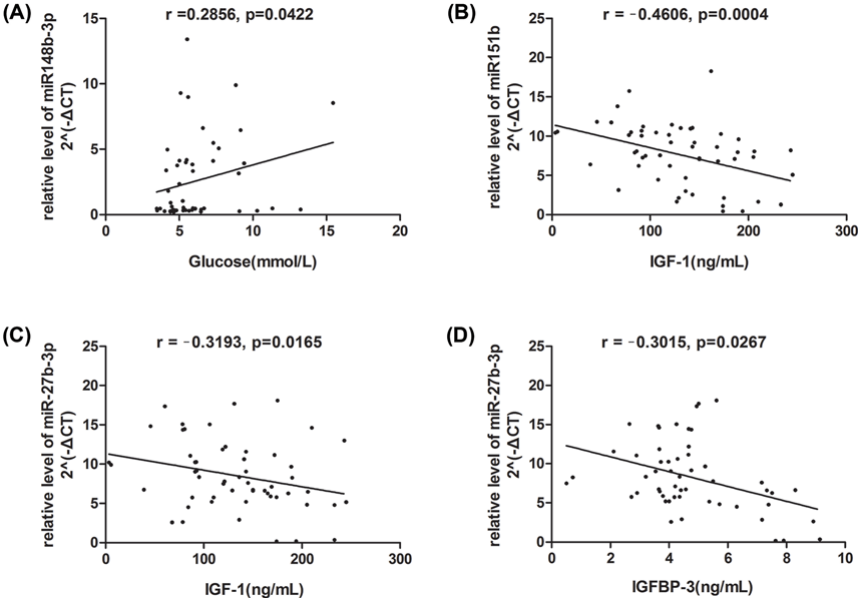
Correlations between miRNA expressions and biochemical parameters The correlation was evaluated by the Spearman test. IGF-1, insulin-like growth factor-1; IGFBP-3, insulin-like growth factor binding protein-3.

There was positive correlation between serum miR-148b level and glucose (*r* = −0.2856, *P*=0.0422) (Figure 2A), miR-151b level showed a strong negative correlation with insulin-like growth factor-1 (IGF-1) (*r* = −0.4606, *P*=0.0004) (Figure 2B), and miR-27b-3p level was negatively correlated with IGF-1 (*r* = −0.3193, *P*=0.0165) (Figure 2C) and insulin-like growth factor binding protein-3 (IGFBP-3) (*r* = −0.3015, *P*=0.0267) (Figure 2D), respectively. No significant correlations were observed between each miRNA and other clinical characteristics, including TG, TC, HDL-C, LDL-C, ApoA or ApoB (Table 2).

**Table 2:** Correlations between microRNAs and clinical characteristics

Characteristics	miR-148b-3p		miR-151b		miR-27b-3p	
	Correlation coefficient (*r*)	*P*	Correlation coefficient (*r*)	*P*	Correlation coefficient (*r*)	*P*
**IGF-1**	0.2346	0.0941	−0.4606	0.0004^†^	−0.3193	0.0165*
**IGFBP-3**	0.04819	0.7370	−0.2171	0.1148	−-0.3015	0.0267*
**Glucose**	0.2856	0.0422*	0.07284	0.6007	−0.06983	0.6158
**TG**	−0.03846	0.7887	−0.05207	0.7084	−0.01780	0.8983
**TC**	−0.1983	0.1631	0.1248	0.3684	0.03152	0.8210
**HDL-C**	0.07424	0.6046	0.08010	0.5648	0.07199	0.6049
**LDL-C**	−0.2567	0.0690	0.1373	0.3223	−0.02097	0.8804
**Apo-A**	0.07528	0.5996	0.02407	0.8628	0.05322	0.7023
**Apo-B**	−0.1592	0.2645	0.03832	0.7833	−0.05669	0.6839
**Apo-B/ Apo-A**	−0.2253	0.1120	0.01907	0.8911	−0.05793	0.6773

Abbreviations: IGF-1, insulin-like growth factor-1; IGFBP-3, insulin-like growth factor binding protein-3. Other abbreviations were shown in Table 1; *P<0.05, ^†^*P*<0.01.

### Diagnostic value of miR-148b-3p, miR-151b and miR-27b-3p levels

**Figure 3:**
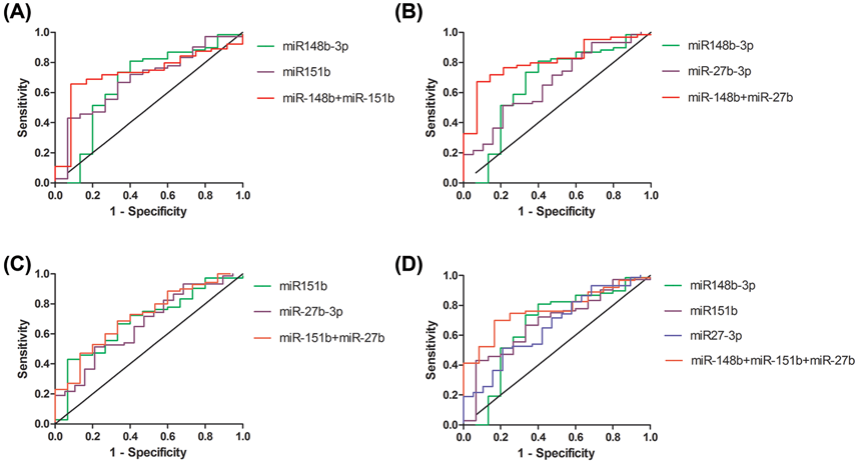
Receiver operating characteristic analysis of miR-148b-3p, miR-151b and miR-27b-3p for ischemic stroke Green, purple and blue lines represent one kind of miRNAs, while red lines signify the combination of two or three miRNAs. The combination of each group was to find out which combinatorial group would have a more powerful value in diagnosing ischemic stroke.

ROC analysis was performed to assess the possibility that circulating miR-148b-3p, miR-151b and miR-27b-3p can serve as potential biomarkers in diagnosing ischemic stroke (Figure 3 and Table 3). Data showed that AUC of miR-148b-3p, miR-151b and miR-27b-3p were 0.6647 (95% CI: 0.4895–0.8399, *P*<0.05), 0.6852 (95% CI: 0.5412–0.8291, *P*<0.05) and 0.6657 (95% CI: 0.5306–0.8008, *P*<0.05) (Table 3), respectively. While the AUC increased when combined with two or three miRNAs, especially when combined miR-148b-3p with miR-27b-3p, the AUC increased to 0.8103 (95% CI: 0.7006–0.9199, *P*<0.001) with a sensitivity and specificity to be 0.6719 and 0.9286, respectively (Figure 3B). Combining miR-148b with miR-151b, the AUC was 0.7266 (95% CI: 0.5856–0.8675, *P*<0.05) with a sensitivity and specificity to be 0.6563 and 0.9167, respectively (Figure 3A) as well as combining miR-151b with miR-27b-3p, the AUC was 0.7143 (95% CI: 0.5763–0.8522, *P*<0.01) with a sensitivity and specificity to be 0.2714 and 0.9333, respectively (Figure 3C). When combined all of three miRNAs, the AUC was 0.7712 (95% CI: 0.6520–0.8903, *P*<0.01) with a sensitivity and specificity to be 0.5238 and 0.9167, respectively (Figure 3D).

**Table 3:** ROC analysis of miRNAs for ischemic stroke

miRNAs	AUC	95% CI	*P* value	Sensitivity	Specificity
**miR-148b-3p**	0.6647	0.4895–0.8399	0.04684*	0.5147	0.8000
**miR-151b**	0.6852	0.5412–0.8291	0.02466*	0.4306	0.9333
**miR-27b-3p**	0.6657	0.5306–0.8008	0.02645*	0.5000	0.7895
**miR-148b+miR-151b**	0.7266	0.5856–0.8675	0.01322*	0.6563	0.9167
**miR-148b+miR-27b-3p**	0.8103	0.7006–0.9199	0.000298^†^	0.6719	0.9286
**miR-151b+ miR-27b-3p**	0.7143	0.5763–0.8522	0.009521^†^	0.2714	0.9333
**miR-148b+miR-151b+ miR-27b-3p**	0.7712	0.6520–0.8903	0.003065^†^	0.5238	0.9167

Abbreviations: AUC, area under the curve; CI, confidence interval; *P<0.05, ^†^*P*<0.01

## Discussion

Currently, no blood biomarkers may be used to predict and diagnose ischemic stroke. Considerable effort has been made to the study of circulating miRNA as biomarkers of kinds of diseases including ischemic stroke since circulating miRNAs were identified in human plasma and serum. Several reports have demonstrated that circulating serum miRNAs could serve as potential non-invasive biomarkers for ischemic stroke. Previously, Yang et al. [8] reported that the circulating levels of miR-107, -128b and -153 were significantly elevated in ischemic stroke patients comparing to healthy controls. He et al. [9] identified 16 differentially expressed miRNAs in the peripheral blood of IS patients, among which miR-145 and miR-122 were the most significantly up-regulated and down-regulated, respectively. In our previous study, Wang et al. [10] explored the diagnostic value of circulating serum miR-221-3p, -382-5p and -4271 in ischemic stroke, and demonstrated that serum miR-221-3p and miR-382-5p could be as potential non-invasive biomarkers for the diagnosis of ischemic stroke. Recently, Tiedt et al. [11] reported circulating miR-125a-5p, miR-125b-5p and miR-143-3p as potential biomarkers in the diagnosis of acute ischemic stroke. However, none of them has been used in clinical practice, the clinical values of all of these miRNAs in IS diagnosis are need to be further investigated. Meanwhile, the standardization of sample processing and detection methods needs to be established.

In the present study, serum levels of miR-148b-3p, miR-151b and miR-27b-3p were the first time to be examined in IS patients. We observed that serum levels of miR-151b and miR-27b-3p in ischemic stroke patients were significantly increased compared with those in healthy controls and the serum levels of miR-148b-3p in ischemic stroke was significantly decreased in comparison with that in healthy controls. MiR-148b-3p belongs to miR-148/152 family, which has been elucidated to be involved in various biological processes [12]. Wang et al. [13] reported that miR-148b regulated proliferation and differentiation of neural stem cell after ischemic stroke. Lehmann et al. [14] reported that miR-27b/miR-17 could discriminate breast cancer with different tumor grades and may be potential molecular markers. Recently, Deng et al. [15] reported that the differential expression of long non-coding RNA in peripheral blood mononuclear cells could serve as biomarker for the diagnosis of acute ischemic stroke, this was an interesting study on the discovery of biomarkers for ischemic stroke diagnosis.

IGF-1 performs a key role in the brain development and normal growth [16]. It reduces the progression of atherosclerosis, and in cross-sectional studies, IGF-1 levels were inversely associated with ischemic stroke, higher serum levels of IGF-1 at the time of stroke is associated with a significant better outcome [17,18]. The activity of IGF-1 is tightly controlled by a family of transportation proteins called insulin-like growth factor-binding proteins (IGFBPs). IGFBP-3/IGF-1 complex represents approximately 70–90% of all circulating IGF-1 levels. They are always investigated concomitantly due to their positive correlation in physiological function [19]. In the present study, the elevation of serum miR-27b-3p was negatively correlated with IGF-1/ IGFBP3, and miR-151b was also in negative correlation with IGF-1. These data were consistent with the above previous findings that miR-151b and miR-27b-3p were sensitive biomarkers upon ischemic stroke with a relatively low expression of IGF-1/IGFBP3. The two type miRNAs together with IGF-1/IGFBP3 may contribute to the progression of ischemic stroke, which remains for further investigation. Besides, we found that serum levels of miR-148b in ischemic stroke patients were positively correlated with blood glucose, and the significance of this correlation needs for future study.

Furthermore, to evaluate the diagnostic value of these three serum circulating miRNAs for IS, we calculated the AUC of serum miR-148b-3p, miR-151b, miR-27b-3p and coupling two or three of them. We found that AUC values of miR-148b-3p, miR-151b, miR-27b-3p were 0.6647, 0.6852 and 0.6657, respectively. Each of them was significantly higher than 0.5 based on statistical analysis, indicating that miR-148b-3p, miR-151b and miR-27b-3p may be favorable for IS diagnosis. Moreover, the more spotlight of the present study was that combination two or three miRNAs had much larger AUC value, especially combination of miR-148b and miR-27b-3p with the largest AUC value 0.8102, and the sensitivity and specificity were 67.19% and 92.86%, respectively. These results strongly indicated that circulating serum miR-148b-3p, miR-151b and miR-27b-3p may be potential non-invasive biomarkers for diagnosing ischemic stroke. And it was more valuable when combined detection of two or three miRNAs than any individual detection of these miRNAs.

The present study has a number of limitations. First, the sample size is relatively small. Second, we tested the change of miRNAs only at the acute phase of stroke, we did not follow in the chronic phase and unable to evaluate the prognostic value of these three miRNAs. Besides, it is worth noticing that although many miRNAs have been reported to be diagnostic for ischemic stroke, there is heterogeneity among individual miRNAs in different studies and none of these reported miRNAs have been put into clinical practice. More researches are required in larger patient populations to further evaluate the diagnostic potential of miRNAs.

## Conclusions

Our findings show down-regulation of serum miR-148b-3p and up-regulation of serum miR-151b and miR-27b-3p in acute IS patients compared with healthy controls and they may serve as blood-based biomarkers for diagnosing ischemic stroke patients, and the combination of miR-148b-3p and miR-27b-3p may be more powerful.

## Abbreviations

ApoA: apolipoprotein A
ApoB: apolipoprotein B
AUC: area under the ROC curve
CT: computed tomography
HDL-C: high-density lipoprotein cholesterol
IGF-1: insulin-like growth factor-1
IGFBP-3: insulin-like growth factor binding protein-3
IS: ischemic stroke
LDL-C: low-density lipoprotein cholesterol
miR: microRNA
MRI: magnetic resonance imaging
ROC: receiver operating characteristic curve
TC: total cholesterol
TG: triglyceride
